# Mental health interventions for carers of individuals with eating disorders: a systematic review of randomized controlled trials assessing efficacy on anxiety and depressive symptoms

**DOI:** 10.1186/s40337-026-01705-8

**Published:** 2026-07-14

**Authors:** Ilaria Riboldi, Chiara Alessandra Capogrosso, Cristina Crocamo, Alessandro Chinello, Luigi Enrico Zappa, Francesco Bartoli, Giuseppe Carrà

**Affiliations:** 1 School of Medicine and Surgery, University of Milano-Bicocca, via Cadore 48, 20900 Monza, Italy; 2 18.56 Monitor Lab, Fondazione Maria Bianca Corno, via Zucchi 1, 20900 Monza, Italy

**Keywords:** Anxiety, Carer, Depression, Eating disorders, Mental health interventions

## Abstract

**Background:**

Carers of individuals with eating disorders (EDs) often experience high levels of psychological distress, which can lead to anxiety and depressive symptoms. While several interventions have been developed to support carers, the effectiveness on their mental health remains unclear.

**Methods:**

We performed a systematic review of randomized controlled trials (RCTs) assessing mental health interventions for carers of individuals with EDs, with anxiety and depressive symptoms defined as outcomes of interest. Searches were performed across major electronic databases up to 31 October 2025.

**Results:**

Twelve RCTs met the inclusion criteria. Structured narrative synthesis indicated that statistically significant effects were infrequent, outcome-specific, and generally small in magnitude. Interventions based on the Cognitive-Interpersonal Maintenance Model (CIMM) were the most frequently evaluated. However, only one trial demonstrated significant improvements in depressive symptoms (p = 0.010), with no significant effect on the Depression Anxiety Stress Scale (DASS-21) total scores (p = 0.06) or on the anxiety subscale (p = 0.50). In addition, Cognitive Behavioural Therapy (CBT)-based interventions showed some promising effects on the Hospital Anxiety and Depression Scale (HADS) total score (p = 0.033), but these results were not consistently replicated across guided and unguided formats. Among the other approaches, a video-based skills-training intervention produced a measurable reduction in carer distress, but this effect was observed only when combined with professional support (p = 0.030). Overall, interventions incorporating professional or peer support appeared more promising than fully self-directed approaches, although direct comparisons between guided and unguided formats did not consistently show statistically significant differences. Quality assessment showed at least an average standard of quality for all the included trials.

**Conclusions:**

Interventions for carers of individuals with EDs are conceptually well-founded, yet current evidence provides only limited support for their effectiveness in reducing anxiety and depressive symptoms. Future research should prioritize interventions that combine more structured mental health strategies with guided self-help programmes and workshops, providing tailored support to address the various needs of carers.

**Supplementary Information:**

The online version contains supplementary material available at https://doi.org/10.1186/s40337-026-01705-8.

## Abstract (plain language summary)

Caring for someone with an eating disorder can be emotionally overwhelming and may affect the carer’s own mental health. This review looked at whether support programmes for carers can help reduce feelings of anxiety, low mood, and emotional strain. We examined studies that tested different kinds of support for carers, including online programmes, workshops, guided self-help, and skills-based training. Overall, these interventions were often helpful in giving carers information, practical strategies, and a better understanding of how to support their loved one. However, the evidence that they improve carers’ own anxiety and depression was limited. Indeed, only a few studies showed significant benefits, and these were usually small, specific to one outcome, or observed only when additional professional support was provided. Programmes that included guidance from a professional or trained supporter seemed more helpful than those completed alone. This suggests that carers may benefit most from support that is not only informative, but also personal and interactive. More research is needed to develop programmes that directly support carers’ mental health, while also helping them care for someone with an eating disorder.

**Supplementary Information:**

The online version contains supplementary material available at https://doi.org/10.1186/s40337-026-01705-8.

## Introduction

Eating disorders (EDs) are severe psychiatric conditions, with a frequent onset during childhood and adolescence, which are developmental stages characterized by psychological vulnerability and strong dependence on carers [1, 2]. Thus, families can play a pivotal role in treatment and recovery, especially, though not exclusively, for younger individuals [3]. As a result, several family-based treatment models have been established as first-line interventions, especially for anorexia nervosa (AN) [4]. However, carers, typically parents and other close relatives, often report feeling unprepared to manage the complex emotional, behavioural, and medical aspects of EDs [5, 6]. In addition, this care-giving role is frequently associated with elevated psychological distress, anxiety and depressive symptoms, which can adversely impact both family functioning and the course of the illness [7–9]. Evidence suggests that several caregiving behaviours, such as overaccommodation, excessive monitoring, or emotional withdrawal, may unintentionally reinforce disordered eating behaviours [10, 11]. Over time, these carer-related patterns can evolve into maladaptive coping strategies such as denial, self-blame, or emotional disengagement, which further exacerbate carer burden and hinder patient recovery [11, 12].

To address these challenges, a growing number of psychosocial interventions have been developed with an additional focus on carers, varying in format, intensity, and psychological orientation [13–15]. Some are grounded in structured theoretical frameworks, such as Cognitive Behavioural Therapy (CBT), or the Cognitive-Interpersonal Maintenance Model (CIMM), while others adopt a more general psychoeducational or skills-based approach**,** including information about ED symptoms and treatment, motivational interviewing or communication skills [13, 16, 17]. In addition, delivery formats can range from unguided self-help materials to structured workshops and therapist-led interventions, with evidence suggesting that more intensive, therapist-guided formats may yield greater improvements in carers’ outcomes [13–15, 18].

Despite the growing body of evidence, carer-oriented interventions remain relatively underdeveloped compared to patient-focused therapies [15]. Although previous reviews have reported positive effects of carer-oriented interventions in EDs on outcomes such as perceived burden, accommodation, self-efficacy, and overall emotional well-being, their specific impact on carers’ anxiety and depressive symptoms remains unclear [7, 19, 20].

To date, no systematic review has specifically examined the efficacy of carer-oriented interventions in reducing anxiety and depression among carers of individuals with EDs. To address this gap, we conducted a systematic review of randomized controlled trials (RCTs) evaluating mental health interventions delivered to carers of individuals with EDs, critically assessing the overall quality of the evidence.

## Material and methods

This systematic review was carried out according to the Preferred Reporting Items for Systematic Reviews and Meta-Analyses (PRISMA) 2020 Statement [21]. A protocol was registered in Open Science Framework Registries on 18 September 2025 (https://doi.org/10.17605/OSF.IO/27YVK).

### Search strategy and inclusion criteria

Systematic searches of Embase, Ovid MEDLINE, and APA PsycInfo databases were performed on 31 October 2025. The search phrase used was: “(psychotherap* OR mindfulness OR self-help OR education OR support OR psychoeducation OR cognitive OR CBT) AND (parent* OR carer* OR caregiver*) AND (anorexi* OR bulimi* OR binge OR eating) AND (intervention OR treatment OR therapy) AND (random* OR controlled OR RCT)” as a multi-purpose search of title, abstract, heading words, and keywords. No language or time restrictions were applied.

We included RCTs comparing any type of mental health interventions, i.e., interventions for mental health support, prevention, and treatment, and an appropriate control condition (e.g., wait-lists or psychoeducational/behavioural interventions or treatment as usual, TAU) for carers of individuals with EDs; i.e., AN, bulimia nervosa (BN), and eating disorder not otherwise specified (EDNOS). To be eligible, RCTs had to report efficacy outcomes for anxiety and/or depressive symptoms as either the between-group difference at the study endpoint or the between-group difference in change from baseline. To improve the consistency and comparability of data, we excluded: (i) studies with a non-randomized and/or non-controlled design; (ii) studies evaluating interventions that focused exclusively on outcomes for individuals with EDs; (iii) “grey” literature, conference abstracts, dissertations, and all publications having not undergone a peer-review process.

After a preliminary screening based on titles and abstracts, full texts were retrieved to evaluate eligibility. Articles were independently screened and read in full text by three authors (I.R., C.A.C., A.C.), and reasons for exclusion were recorded (see Additional file 1). Any disagreement was resolved by discussion with the other authors.

### Data extraction

Three authors (I.R., C.A.C., A.C.) independently extracted the data and performed blind cross-checks to ensure accuracy. A standardized data extraction template was used to collect key information from the included studies, such as author(s) and year of publication, country, study setting, design, main characteristics of experimental (EG) and control groups (CG), type of EDs, and level of care (i.e., outpatient, day treatment, or inpatient services). Details on assessment tools, measured outcomes, and corresponding metrics were also collected. Effect size measures were extracted (e.g., Cohen’s d, partial eta-squared, and regression coefficients standardised by the baseline standard deviations of the outcome) and classified using well-established thresholds [22, 23]. To improve interpretability, we also extracted the direction of effect, statistical significance, and whether the reported effect could reasonably be interpreted as clinically meaningful, considering the magnitude of the effect and the availability of clinically significant change thresholds.

### Data synthesis

A quantitative synthesis was initially considered; however, meta-analysis was deemed inappropriate because of the substantial clinical and methodological heterogeneity among the eligible studies. First, the outcome measures differed markedly across trials. Second, the interventions varied in their theoretical underpinnings, intensity, mode of delivery, level of guidance, and comparator conditions, encompassing unguided self-help materials, guided online programmes, workshops, video-based skills training, treatment as usual, and psychoeducational controls. Third, studies reported findings using heterogeneous statistical approaches and assessment time points, including endpoint between-group comparisons and pre–post change scores. Consequently, only a very limited number of studies were sufficiently comparable to support any meaningful pooled contrast, and statistical pooling would have yielded summary estimates of limited clinical interpretability. We therefore conducted a structured narrative synthesis, organized according to intervention type and delivery format, supplemented by vote-counting based on the direction and statistical significance of effects and by descriptive summaries of effect-size magnitude.

### Quality assessment of the included studies

The quality of the studies included in the systematic review was formally assessed using the Randomized Controlled Trial of Psychotherapy Quality Rating Scale (RCT-PQRS), a validated 25-item tool specifically developed to evaluate the methodological rigor of psychotherapy RCTs [24, 25]. This instrument was selected because the included interventions were psychosocial, psychotherapy-informed, or skills-based. The RCT-PQRS was considered appropriate as it assesses key methodological domains relevant to complex behavioural trials, including treatment specification, facilitator training, intervention fidelity, outcome assessment, statistical analysis, and reporting quality [24, 25]. Items from 1 to 24 assess key domains such as participant characteristics, definition and allocation of treatment/intervention, outcome measures, data analysis, and reporting procedures, each scored on a scale of 0 to 2. Item 25 provides a global quality rating of the study, ranging from 1 (“exceptionally poor”) to 7 (“exceptionally good”). A total score of 24 or higher on the first 24 items, equivalent to an average score of 1 per item, is considered the minimum threshold for acceptable study quality [25]. Three authors (I.R., C.A.C., and A.C.) independently evaluated the included studies, with any discrepancies resolved through discussion among all authors.

## Results

### Study selection

**Fig. 1:**
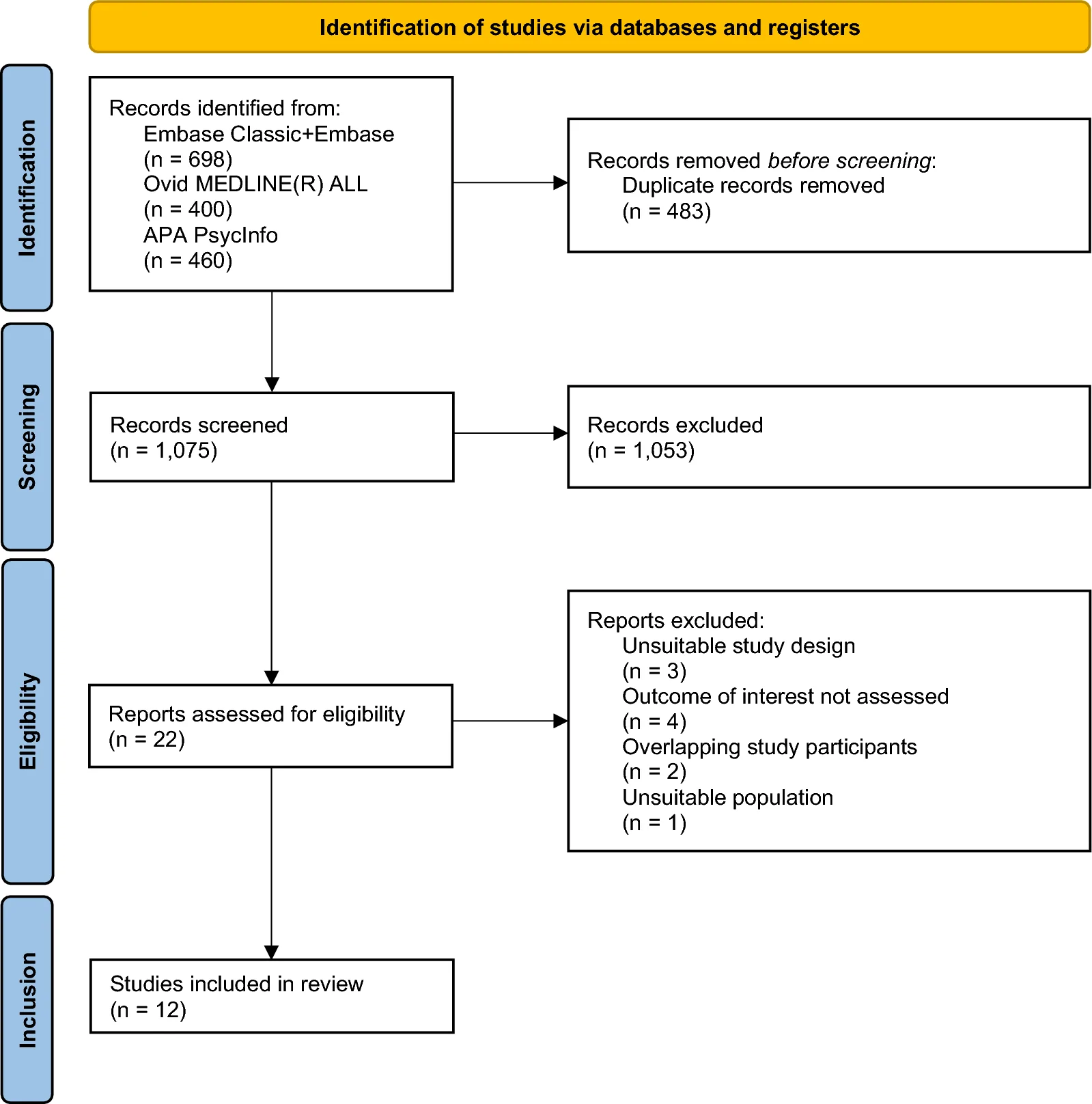
Flowchart of the systematic review according to PRISMA criteria

As a whole, our systematic search generated 1,558 records (698 from Embase, 400 from Ovid MEDLINE, and 460 from APA PsycInfo), reduced to 1,075 unique articles after deduplication. After the screening of titles and abstracts, 22 articles were retrieved and examined in full text. Among them, 12 studies [26–37] met the inclusion criteria and were included. The study selection process is fully described in Fig. 1.

### Study characteristics

Twelve RCTs comprising 1,709 carers were included. Participants were primarily parents or other non-professional carers of individuals mostly diagnosed with anorexia nervosa (AN), recruited across a variety of treatment settings, including inpatient care. The interventions differed in theoretical foundation, delivery format, and intensity, and were grouped into three main categories: (i) structured CIMM-based interventions [26, 27, 29, 30, 32, 35–37]; (ii) CBT-based interventions [28, 31]; (iii) other interventions [33, 34]. Delivery formats included a range of approaches, from self-help resources such as manuals, videos, and digital platforms to therapist- or peer-led self-help sessions and guided workshops. The duration was generally limited, with no booster sessions provided due to the nature of the interventions. Control conditions typically consisted of waitlists, TAU, or alternative psychoeducational interventions. However, it is important to note that, in two studies, the control group received the same intervention as the EG, but without guidance [27, 31]. All studies assessed anxiety and depressive symptoms in carers based on validated scales, either as separate domains or combined into a total score.

Main study characteristics are presented in Table 1, with detailed descriptions of the included interventions reported in Additional file 2.

**Table 1:** Characteristics of the included studies

Study	Country	N tot	N EG	N CG	Female gender (%)	Target group	EG intervention	CG intervention	Outcomes	Assessment tools
Cardi et al., 2024	UK	371	185	186	259 (70.0%)	Family, partners, friends of individuals with AN or atypical AN (> = 16 y), in-/day-patient	ECHOMANTRA (guided) + TAU	TAU	Anxiety and depression	DASS-21 total score
Goddard et al., 2011	UK	153	73	80	136 (88.9%)	Family and friends providing unpaid support to individuals with AN, BN, or EDNOS across all treatment levels	ECHO guided	ECHO (unguided)	Anxiety and depression	HADS total score; GHQ-12
Grover et al., 2011	UK	63	33	30	N/R	Relatives, partners, and friends of individuals with broadly defined AN, across all treatment levels	OAO-G	Support	Anxiety and depression	HADS total score
Hibbs et al., 2015	UK	268	134	134	161 (60.1%)	Family or friends of individuals > 12 years with AN, day- or in-patients	ECHO (guided) + TAU	TAU	Anxiety and depression	DASS-21 total score
Hodsoll et al., 2017	UK	226	72 (ECHO); 78 (ECHOg)	76	155 (68.6%)	Parents of individuals aged 13–20 years with a primary diagnosis of AN or atypical AN, outpatient services	ECHO (unguided) + TAU; ECHO (guided) + TAU	TAU	Anxiety and depression	DASS-21 total score
Hoyle et al., 2013	Australia/UK	37	19	18	33 (89.1%)	Family members of individuals with AN	OAO-G	OAO-NoG	Anxiety and depression	DASS-21 total score; GHQ-28
Keshen et al., 2020	Canada	48	25	23	24 (50.0%)	Family and friends of individuals with ED, outpatient services	ECHO (unguided) + TAU	TAU	Anxiety and depression	DASS-21 total score
McEvoy et al., 2019	Australia	33	18	15	20 (61.0%)	Family, partners, and friends of individuals with AN, atypical AN, or BN; no current formal diagnosis required	Brief psychoeducation and communication skills training	Waitlist	Anxiety; Depression	PROMIS-A; PROMIS-D
Quadflieg et al., 2017	Germany	285	147; 38 (EG +)	138; 36 (CG +)	N/R	Daily care provider of individuals with AN, BN, EDNOS, or atypical variants, following at least 4 weeks of inpatient treatment	Video-based intervention based on CIMM; EG + : plus additional professional support	TAU; CG + : plus additional professional support	Anxiety and depression	GHQ-12
Quiles Marcos et al., 2018	Spain	64	40	24	38 (59.4%)	Individuals currently living with a person with AN, BN, or EDNOS, outpatients	CCSTW	Psychoeducation	Anxiety; Depression	HADS-A; HADS-D; GHQ-12
Ruiz et al., 2026	Spain	108	54	54	92 (85.0%)	Primary carers of individuals diagnosed with AN, BN, or EDNOS during inpatient, day-patient, or outpatient treatment	ECHOMANTRA (guided) + TAU	TAU	Anxiety; Depression	DASS-21 total score; DASS-21-A; DASS-21-D
Sepúlveda et al., 2019	Spain	53	27	26	52 (98%)	Individuals directly involved in the care of a relative with AN, BN, or EDNOS, treated in outpatient services	CCSTW	Psycho-educational workshop	Anxiety; Depression	HADS-A; HADS-D; GHQ-12

CG = control group; EG = experimental group; AN = anorexia nervosa; BN = bulimia nervosa; EDNOS = eating disorders not otherwise specified; ED = eating disorder

CIMM = Cognitive Interpersonal Maintenance Model; ECHO = Experienced Carers Helping Others; ECHOMANTRA = Experienced Carers Helping Others + Maudsley Model of Anorexia Nervosa Treatment for Adults; OAO-G = Overcoming Anorexia Online guided; OAO-NoG = Overcoming Anorexia Online no guidance; CCSTW = Collaborative Care Skills Training Workshops; TAU = treatment as usual; N/R = not reported

### Study findings

Table 2 shows a summary of findings of the effects of interventions on carers’ anxiety and depressive symptoms.

**Table 2:** Summary of findings of interventions

Study	Intervention / comparator	Outcome domain	Main finding	Statistical result / effect size	Clinical interpretation
Cardi et al., 2024	Guided ECHOMANTRA + TAU vs TAU	Overall anxiety/depression-related distress	No between-group difference at endpoint in DASS-21 total scores at 12 or 18 months	DASS-21 total: d = -0.06 (12 months), p = 0.57; d = -0.09 (18 months), p = 0.43; negligible	No evidence of clinically meaningful improvement in overall distress. Anxiety- and depression-specific effects cannot be inferred separately from the total score
Goddard et al., 2011	Guided ECHO vs unguided ECHO	Combined anxiety/depressive symptoms	No pre-post mean change difference in HADS total scores between EG and CG	HADS total: standardized effect size N/R; p-value N/R	No clear incremental benefit of guidance for combined anxiety/depressive symptoms
	Guided ECHO vs unguided ECHO	General psychological distress	No pre-post mean change difference in GHQ-12 total scores between EG and CG	GHQ-12: effect size N/R; p = 0.30	Improvement, if present, may reflect time, shared intervention content, or non-specific effects rather than guidance-specific clinical benefit
Grover et al., 2011	Guided OAO vs ad hoc support	Combined anxiety/depressive symptoms	Greater pre-post mean reduction in HADS total score in EG compared with CG	HADS total: MD = -3.8 (95% CI -7.3 to -0.3), p = **0.033**	Potentially meaningful reduction in combined symptoms, but clinical significance cannot be confirmed because reliable change thresholds were not reported
Hibbs et al., 2015	Guided ECHO + TAU vs TAU	Overall anxiety/depression-related distress	No between-group advantage at post-intervention, 6 months, or 12 months on DASS-21 total score	DASS-21 total: ES = -0.02, p = 0.86; 6 months ES = -0.17, p = 0.16; 12 months ES = -0.12, p = 0.33; negligible to small	No evidence that guided ECHO reduced overall distress beyond TAU. Separate anxiety/depression effects were not available
Hodsoll et al., 2017	ECHO overall + TAU vs TAU; guided vs unguided ECHO	Overall anxiety/depression-related distress	No endpoint difference for ECHO overall vs TAU, nor for guided vs unguided ECHO, on DASS-21 total score	DASS-21 total: ES = 0.07, p = 0.72 (ECHO vs TAU); ES = 0.23, p = 0.31 (guided vs unguided); negligible to small	Effects were small and statistically non-significant; no robust evidence of clinically relevant improvement in overall distress
Hoyle et al., 2013	Guided OAO vs unguided OAO	Overall anxiety/depression-related distress / general psychological distress	No pre-post mean change difference between EG and CG in DASS-21 total score or GHQ-28 total score	Standardized effect size N/R; p-value N/R	Adding optional guidance did not clearly improve overall distress in this small trial; anxiety- and depression-specific effects were not separately reported
Keshen et al., 2020	Unguided ECHO DVD + TAU vs TAU	Overall anxiety/depression-related distress	Numerically favoured EG, but no significant between-group advantage on DASS-21 total score at endpoint or follow-up	DASS-21 total: d = 0.23, p > 0.05; 3 months d = 0.34, p > 0.05; small	Small non-significant effects suggest limited clinical impact of standalone DVD self-help on overall distress
Quadflieg et al., 2017	Video-based skills training vs TAU; subgroup with additional professional support	General psychological distress	No overall effect on GHQ-12; significant benefit only in subgroup receiving additional professional support	GHQ-12 overall: partial eta-squared = 0.01, p = 0.64; supported subgroup: partial eta-squared = 0.07, p = **0.030**; moderate in supported subgroup	Professional support may moderate benefit. Video materials alone did not show clinically meaningful benefit
McEvoy et al., 2019	Brief group psychoeducation/communication skills vs waitlist	Anxiety symptoms	No between-group difference at endpoint in PROMIS-A scores	PROMIS-A: d = 0.41, p > 0.05; follow-up d = 0.34, p > 0.05; small to approaching moderate	Numerical improvement in anxiety was not statistically significant; clinical benefit remains uncertain, particularly given sample size and lack of reliable change thresholds
	Brief group psychoeducation/communication skills vs waitlist	Depressive symptoms	No between-group difference at endpoint in PROMIS-D scores	PROMIS-D: d = 0.28, p > 0.05; follow-up d = 0.30, p > 0.05; small	No statistically supported depression-specific benefit; observed effects were small and clinically uncertain
	CCSTW vs psychoeducation	General psychological distress	No pre-post mean change difference in GHQ-12 scores between EG and CG	GHQ-12: standardized effect size N/R	Psychological distress improved over time, but no between-group advantage over psychoeducation was demonstrated
Quiles Marcos et al., 2018	CCSTW vs psychoeducation	Anxiety symptoms	No pre-post mean change difference in HADS-A scores between EG and CG	HADS-A: standardized effect size N/R	No anxiety-specific advantage over psychoeducation was demonstrated
	CCSTW vs psychoeducation	Depressive symptoms	No pre-post mean change difference in HADS-D scores between EG and CG	HADS-D: standardized effect size N/R	Depressive symptoms improved over time, but no between-group advantage over psychoeducation was demonstrated
	Guided online ECHOMANTRA + TAU vs TAU	Overall anxiety/depression-related distress	No statistically significant time-by-group effect on DASS-21 total score	DASS-21 total: F(4) = 2.26, p = 0.06; standardized effect size N/R	A trend in overall distress did not reach statistical significance; clinical magnitude remains uncertain
Ruiz et al., 2026	Guided online ECHOMANTRA + TAU vs TAU	Anxiety symptoms	No between-group difference in DASS-21 anxiety scores	DASS-21-A: F(4) = 0.85, p = 0.50; standardized effect size N/R	No anxiety-specific treatment effect was demonstrated
	Guided online ECHOMANTRA + TAU vs TAU	Depressive symptoms	Significant time-by-group effect favouring EG for DASS-21 depression scores	DASS-21-D: F(4) = 3.15, p = **0.010**; standardized effect size N/R	Possible depression-specific benefit, but clinical magnitude cannot be established without standardized effect sizes or clinically meaningful change thresholds
	CCSTW vs psychoeducational workshop	General psychological distress	No between-group difference at endpoint in GHQ-12 total score	GHQ-12: partial eta-squared = 0.011, p = 0.59; small	No evidence of clinically meaningful improvement in general psychological distress beyond psychoeducation
Sepúlveda et al., 2019	CCSTW vs psychoeducational workshop	Anxiety symptoms	No between-group difference at endpoint in HADS-A scores	HADS-A: partial eta-squared = 0.035, p = 0.19; small	Despite the reported effect estimate, the anxiety effect was not statistically significant; clinical benefit is therefore unsupported and should be interpreted cautiously
	CCSTW vs psychoeducational workshop	Depressive symptoms	No between-group difference at endpoint in HADS-D scores	HADS-D: p = 0.87; standardized effect size N/R	No depression-specific clinical benefit beyond psychoeducation was demonstrated

CG = control group; EG = experimental group; FU = follow-up; TAU = treatment as usual; N/R = not reported; MD = mean difference; ES = effect size; CI = confidence interval

HADS = Hospital Anxiety and Depression Scale; HADS-A = Hospital Anxiety and Depression Scale-Anxiety; HADS-D = Hospital Anxiety and Depression Scale-Depression; DASS-21 = Depression Anxiety Stress Scale-21; DASS-21-A = DASS-21-Anxiety; DASS-21-D = DASS-21-Depression; GHQ = General Health Questionnaire; PROMIS-A = Patient-Reported Outcomes Measurement Information System-Anxiety; PROMIS-D = Patient-Reported Outcomes Measurement Information System-Depression

Effect-size interpretation: Cohen's d/standardized ES approximately 0.20 = small, 0.50 = moderate, 0.80 = large; partial eta-squared approximately 0.01 = small, 0.06 = moderate, 0.14 = large. Clinical interpretation was based on statistical significance, magnitude of effect, and whether clinically significant change or threshold-based improvement was reported

Where studies reported only total scores (e.g., DASS-21 total, HADS total, GHQ total), findings are presented as overall anxiety/depression-related or general psychological distress rather than as anxiety- or depression-specific effects

Overall, the magnitude of effects across studies was generally negligible to small. Large effects were not observed. Moderate effects were rare and limited to specific contexts, particularly the subgroup receiving additional professional support in the video-based skills-training intervention. Small-to-approaching-moderate effects were reported in some trials, but these were often statistically non-significant or not consistently replicated. Therefore, the available evidence suggests that, when present, effects on carers’ anxiety and depressive symptoms were generally limited in magnitude and of uncertain clinical significance.

### Structured CIMM-based interventions

Eight studies evaluated structured interventions based on the CIMM, a model that explains how cognitive, emotional, and interpersonal factors interact to maintain eating disorders, with a primary focus on addressing unhelpful caregiving behaviours and maladaptive communication patterns [38, 39]. The included interventions comprised Experienced Carers Helping Others (ECHO), ECHOMANTRA, and Collaborative Care Skills Training Workshops (CCSTW). ECHO is a carer-focused psychoeducational and skills-based programme designed to help carers understand and manage interpersonal factors involved in the maintenance of anorexia nervosa [40], originally delivered through workshops aimed at reducing carer distress [41]. ECHOMANTRA combines ECHO for carers with the Maudsley Model of Anorexia Nervosa Treatment for Adults (MANTRA) for patients [42]. CCSTW is a structured group intervention grounded in the CIMM, combining psychoeducation with practical strategies such as motivational interviewing, problem-solving, and communication skills to reduce expressed emotion and support behaviour change [43].

Overall, CIMM-based interventions showed limited evidence of efficacy in reducing carers’ anxiety and depressive symptoms compared with treatment as usual, unguided formats, or psychoeducational controls. Several ECHO-based trials reported no significant between-group effects on combined measures of anxiety, depression, or psychological distress.

More specifically, in 2011, 153 carers were allocated to phone-guided ECHO (N = 73) or self-help–only ECHO (N = 80). Although HADS scores decreased post-intervention in both groups (p < 0.001), no significant between-group difference was found for anxiety/depression symptoms or GHQ-12 psychological distress (p = 0.30) [27]. Similarly, Hibbs et al. [29] compared guided ECHO plus TAU with TAU alone in 134 carers per group and found no significant between-group difference on DASS-21 total scores at endpoint (effect size =  − 0.02; p = 0.86). In another trial including 226 carers of individuals aged 13–20 years receiving outpatient care, neither overall ECHO versus TAU (effect size = 0.07; p = 0.72) nor guided versus unguided ECHO (effect size = 0.23; p = 0.31) showed significant differences in DASS-21 total scores at 12 months [30]. Finally, Keshen et al. [32] evaluated a self-help ECHO DVD in an outpatient setting, comparing standard treatment alone (N = 23; up to 32 weeks, four days per week) with standard treatment plus a four-week ECHO DVD intervention (N = 25). No significant endpoint difference was observed on DASS-21 total scores (d = 0.23; p > 0.05).

Evidence from ECHOMANTRA trials was also mixed. In the largest trial, Cardi et al. [26] randomized carers to ECHOMANTRA plus TAU (N = 185) or TAU alone (N = 186). The intervention included digital self-management materials and eight group sessions for patients, carers, and dyads. No significant between-group differences were observed in DASS-21 total scores at 12 months (d =  − 0.06; p = 0.57) or 18 months (d =  − 0.09; p = 0.43), with negligible effect sizes. Conversely, Ruiz et al. [36] evaluated online ECHOMANTRA in 108 carers randomized to TAU or TAU plus eight weekly individual online sessions delivered by trained assistant psychologists. At endpoint, the intervention group showed a significantly greater reduction in DASS-21 depression scores (p = 0.010), but no significant differences in DASS-21 total scores (p = 0.06) or anxiety symptoms (p = 0.50). Overall, these findings suggest a possible depression-specific benefit in one trial, but no consistent effect on overall distress or anxiety.

Evidence for CCSTW was also limited with regard to carers’ anxiety and depressive symptoms. Quiles Marcos et al. [35] assessed CCSTW in 40 carers and found a significant decrease over time in psychological distress and depression (p = 0.045), but no significant between-group differences for HADS anxiety, HADS depression, or GHQ-12 scores compared with psychoeducation alone. Similarly, Sepúlveda et al. [37] evaluated CCSTW in 27 carers and found no significant between-group differences in GHQ-12 psychological distress (ηp^2^ = 0.011; p = 0.59), HADS anxiety (ηp^2^ = 0.035; p = 0.19), or depressive symptoms (p = 0.87). Taken together, these findings suggest that CIMM-based interventions may provide useful psychoeducational and caregiving support, but current RCT evidence provides limited support for a robust effect on carers’ anxiety and depressive symptoms.

### CBT-based interventions

Two studies assessed Overcoming Anorexia Online (OAO), a web-based programme grounded in CBT principles, emphasizing shared formulation and reciprocal influences between family members’ thoughts, emotions, and behaviours [28, 31]. Grover et al. [28] randomized 64 carers to OAO with limited clinician support or ad hoc support from a UK patient-carer organization. The intervention group showed a significantly greater pre–post reduction in combined anxiety and depressive symptoms than controls, as measured by the HADS total score (mean difference =  − 3.8; p = 0.033). Conversely, Hoyle et al. [31] compared guided OAO (OAO-G) with unguided OAO (OAO-NoG) in 37 carers. Both groups completed seven online modules and two workbooks over seven weeks, with optional weekly guidance in the OAO-G arm. No between-group differences in change from baseline were observed on DASS-21 or GHQ-28 scores. Overall, CBT-based digital interventions appear promising, particularly when some clinician support is provided, but current evidence remains limited and not consistently replicated across guided and unguided formats*.*

### Other interventions

Other interventions included adaptations of the broader CIMM framework, delivered through workshop-based or digital skills-training formats. Quadflieg et al. [34] randomized 285 carers from inpatient settings to a video-based skills-training intervention (N = 147) or TAU (N = 138), with a subgroup in each arm also receiving professional support through psychotherapy or clinical counselling (EG N = 38; CG N = 36). No significant difference was found in GHQ-12 scores in the overall sample (ηp^2^ = 0.01; p = 0.64), whereas a significant moderate effect emerged in the professionally supported subgroup (ηp^2^ = 0.07; p = 0.03). McEvoy et al. [33] evaluated a brief face-to-face New Maudsley Method intervention [44], consisting of two 2.5-h group sessions delivered one week apart versus waitlist control in 44 carers. No significant effects were observed on PROMIS anxiety or depression at post-intervention or follow-up, although small-to-approaching-moderate effects numerically favoured the intervention for PROMIS-A (d = 0.41 post; d = 0.34 follow-up; p > 0.05) and PROMIS-D (d = 0.28 post; d = 0.30 follow-up; p > 0.05). Overall, these findings suggest that brief skills-based interventions may require sufficient intensity and professional support to produce clinically robust effects.

### Quality assessment

The quality assessment of the included studies, performed using the RCT-PQRS, resulted in scores ranging from 29 to 40 (mean = 35.5; standard deviation (SD) = 3.3). Based on the established cut-off of 24 points, this indicates that all trials met at least average standards of quality, with several achieving a very good level [25]. Outcome measures (i.e., outcome assessment by raters blinded to treatment group and with established reliability), and discussion of safety and adverse events during study treatment were among the domains rated with the lowest score. The complete quality assessment is reported in Table 3.

**Table 3:** Quality assessment of the included studies using the Randomized Controlled Trial of Psychotherapy Quality Rating Scale

Author(s), year	RCT-PQRS items																									
	*1*	*2*	*3*	*4*	*5*	*6*	*7*	*8*	*9*	*10*	*11*	*12*	*13*	*14*	*15*	*16*	*17*	*18*	*19*	*20*	*21*	*22*	*23*	*24*	***Total***^*†*^	*25*^***‡***^
Cardi et al., 2024	2	2	2	2	2	1	1	0	2	2	2	2	0	2	2	1	2	2	0	2	2	2	2	2	39	6
Goddard et al., 2011	2	1	2	2	2	1	2	2	2	2	2	0	0	1	0	0	1	2	0	2	2	2	2	2	34	5
Grover et al., 2011	2	1	2	2	2	1	2	2	2	2	2	2	0	1	0	1	2	2	0	2	2	2	2	2	38	6
Hibbs et al., 2015	2	2	2	2	2	1	1	0	2	2	2	0	0	2	2	0	2	1	0	2	2	2	2	2	35	6
Hodsoll et al., 2017	2	2	2	2	2	1	1	0	2	2	2	2	0	2	2	0	2	2	0	2	2	2	2	2	38	6
Hoyle et al., 2013	1	1	2	1	1	1	2	0	2	2	2	0	0	1	0	0	2	2	0	2	2	1	2	2	29	4
Keshen et al., 2020	2	2	2	2	2	1	1	0	2	1	2	0	0	1	2	0	2	1	0	2	2	2	2	2	33	5
McEvoy et al., 2019	2	1	2	2	2	2	2	0	2	2	2	2	0	0	1	2	2	2	0	2	2	2	2	2	38	6
Quadflieg et al., 2017	2	2	2	2	2	1	1	0	2	2	2	0	0	1	0	1	2	2	0	2	2	1	2	2	33	5
Quiles Marcos et al., 2018	2	1	2	2	2	1	2	0	2	2	2	2	0	1	2	0	2	2	0	2	2	2	2	2	37	6
Ruiz et al., 2026	2	1	2	2	2	1	2	2	2	2	2	2	0	2	2	1	2	1	0	2	2	2	2	2	40	6
Sepúlveda et al., 2019	2	1	2	2	2	1	2	0	2	2	2	0	0	1	0	0	2	2	0	2	2	1	2	2	32	5

RCT-PQRS = Randomized Controlled Trial of Psychotherapy Quality Rating Scale [24]

^†^The total quality-of-study score is obtained by summing items from #1 to #24 and ranges from 0 to 48. *A total quality-of-study score equal to or greater than 24 corresponds to the minimum adequate study quality* [25]

^**‡**^The omnibus score, which coincides with item #25, ranges from 1 (exceptionally poor study) to 7 (exceptionally good study)

## Discussion

To our knowledge, this is the first systematic review to synthesize evidence from RCTs evaluating mental health interventions specifically designed for carers of individuals with EDs, with a focus on their effects on anxiety and depressive symptoms. Across intervention types, programmes based on the CIMM, particularly ECHO, were the most extensively studied. Despite their strong theoretical basis and widespread implementation, these interventions generally failed to demonstrate significant reductions in carers’ anxiety or depression compared with TAU or psychoeducational controls. An exception was an online ECHOMANTRA trial, which reported significant reductions in depressive symptoms, though without corresponding improvements in general distress and anxiety. In addition, CBT-based approaches, especially OAO, yielded some encouraging findings, with one trial reporting significant improvements in overall anxiety and depression scores. However, subsequent comparisons of guided versus unguided delivery formats did not replicate these effects. Among other approaches, only a video-based intervention seems to have some effect, even though receiving additional external professional help was identified as a moderator contributing to the efficacy of the intervention. Although the included trials were generally of high methodological quality, the available evidence provides only limited support for the effectiveness of the included mental health interventions in reducing anxiety and depression among carers of individuals with EDs. Overall, the examined interventions are theoretically sound and have shown benefits on caregiving-related outcomes such as accommodation, self-efficacy, perceived burden, and general emotional well-being [13, 14, 19, 20]. However, their specific effects on carers’ anxiety and depressive symptoms appear more limited. CIMM- and CBT-based approaches share cognitive foundations and often include psychoeducation, skills training, communication enhancement, and strategies to modify maladaptive responses, but they differ in their primary targets and mechanisms of change [39, 45]. CIMM-based interventions mainly address interpersonal and family-level maintaining factors, aiming to improve communication and reduce conflict, whereas CBT-based approaches focus more directly on maladaptive thoughts, behaviours, coping strategies, and emotional regulation at the individual level [39, 45]. Both frameworks remain relevant to carers’ mental health. The CIMM provides a useful model for understanding how relational and caregiving processes may contribute to distress in the context of EDs [39, 46, 47], while evidence from other caregiving contexts suggests that CBT-based components, including cognitive restructuring, behavioural activation, self-care, and stress management, may reduce anxiety and depressive symptoms among carers [48–51]. Nevertheless, the limited effects observed in the included RCTs suggest that many interventions may have lacked sufficient specificity, intensity, or therapeutic exposure to adequately address internalizing symptoms [52–55]. Consistently, guided interventions appeared more promising than unguided self-help or standalone psychoeducation, in line with evidence that structured support, facilitated sessions, and coaching may enhance engagement, adherence, validation, and self-efficacy [15, 56–58]. Conversely, the absence of guidance may leave carers’ unmet needs, emotional burden, frustration, and feelings of helplessness insufficiently addressed, particularly in the context of the chronic and complex course of EDs [59–61].

From a clinical and service-delivery perspective, these findings indicate that carer-focused interventions should include explicit assessment of carers’ anxiety and depressive symptoms, rather than focusing solely on psychoeducation or caregiving skills [7, 14, 15]. Psychoeducational or skills-based self-help may be appropriate for carers with mild distress, but should preferably include guidance, feedback, and interaction [56, 58]. Conversely, carers with persistent or moderate-to-severe symptoms may require more structured psychological support, including CBT-informed strategies, behavioural activation, stress-management techniques, or referral to formal mental health care [52, 53]. At the service and policy level, carer interventions should be integrated into eating disorder care pathways, supported by facilitator training, and evaluated using outcomes that include carers’ mental health [13–15, 56, 62].

Future research should integrate targeted mental health components into established interventions based on skills training, communication enhancement, and psychoeducation [52, 62]. These programmes, including self-help formats, should be delivered with adequate guidance, intensity, and duration [56]. RCTs should also better characterize carer subgroups, such as siblings living in the same household, whose caregiving exposure and symptom profiles may differ substantially [63, 64]. Finally, given the predominant focus on anorexia nervosa, further research is needed on carers of individuals with other eating disorders, particularly regarding anxiety and depressive symptoms [7, 14].

### Limitations

The findings of this systematic review should be interpreted in light of several limitations. First, substantial heterogeneity was observed across studies regarding design, sampling strategies, participant characteristics, intervention content and duration, assessment tools, outcome definitions, and effect size reporting. Differences in control conditions further complicate interpretation. This variability precluded the possibility of conducting a quantitative meta-analysis and limits direct comparability across trials. Second, blinding was not feasible in most studies due to the nature of the interventions, increasing the risk of performance and detection bias. Third, several included trials had relatively small sample sizes, which may have reduced statistical power and increased the imprecision of effect estimates. In addition, publication bias could not be formally assessed because of the small number of eligible studies and the lack of sufficiently comparable effect estimates. Furthermore, the available data did not allow us to assess whether intervention outcomes varied according to carer characteristics such as gender, age, education level, personal history of EDs and other comorbid mental health diagnoses. Finally, long-term follow-up data were scarce, making it difficult to determine whether any observed benefits were sustained over time or whether periodic reinforcement might be necessary to maintain gains.

## Conclusions

Taken together, our findings support the hypothesis that, although interventions for carers of individuals with EDs are conceptually well-founded and often effective in improving caregiving skills and overall emotional well-being, evidence of their specific impact on carers’ anxiety and depressive symptoms remains limited. Future research should prioritize interventions that combine ED-specific carer skills training with explicit mental health components for carers. Such interventions should provide adequate therapeutic intensity and report standardized, clinically interpretable outcomes to determine whether observed benefits translate into meaningful improvements in routine practice.

## Supplementary Information

Additional file 1.

Additional file 2.

## Data Availability

All data generated or analysed during this study are included in this published article and its supplementary information files.

## Abbreviations

ED/EDs: Eating disorders
RCT/RCTs: Randomized controlled trials
CIMM: Cognitive-Interpersonal Maintenance Model
CBT: Cognitive Behavioural Therapy
DASS-21: Depression Anxiety Stress Scale-21 items
HADS: Hospital Anxiety and Depression Scale
AN: Anorexia nervosa
PRISMA: Preferred Reporting Items for Systematic Reviews and Meta-Analyses 2020 Statement
BN: Bulimia nervosa
EDNOS: Eating disorder not otherwise specified
EG: Experimental group
CG: Control group
TAU: Treatment as usual
RCT-PQRS: Randomized Controlled Trial of Psychotherapy Quality Rating Scale
ECHO: Experienced Carers Helping Others
GHQ-12: General Health Questionnaire-12 items
MANTRA: Maudsley Model of Anorexia Nervosa Treatment for Adults
CCSTW: Collaborative Care Skills Training Workshops
OAO: Overcoming Anorexia Online
OAO-G: Overcoming Anorexia Online-guided
OAO-NoG: Overcoming Anorexia Online-unguided
GHQ-28: General Health Questionnaire-28 items
PROMIS-A: Patient-Reported Outcomes Measurement Information System Anxiety
PROMIS-D: Patient-Reported Outcomes Measurement Information System Depression
SD: Standard deviation
