# Supplementary material for: Mental health interventions for carers of individuals with eating disorders: a systematic review of randomized controlled trials assessing efficacy on anxiety and depressive symptoms
Source: J Eat Disord. 2026 Jul 14;14:235. doi: 10.1186/s40337-026-01705-8 (PMC13647608; doi:10.1186/s40337-026-01705-8)
Supplement: Supplementary file 1 [file 40337_2026_1705_MOESM1_ESM.docx]

# Table A.1. List of the articles excluded after full-text revision with reasons for exclusion.

| **Cluster of exclusion** | **Reference** | **Specific reason for exclusion** |
| --- | --- | --- |
| Unsuitable study design  (3) | Truttmann S, Philipp J, Zeiler M, Franta C, Wittek T, Merl E, et al. Long-Term Efficacy of the Workshop Vs. Online SUCCEAT (Supporting Carers of Children and Adolescents with Eating Disorders) Intervention for Parents: A Quasi-Randomised Feasibility Trial. J Clin Med. 2020;9(6):1912. https://doi.org/10.3390/jcm9061912. | Quasi-randomised feasibility trial. |
|  | Whitney J, Murphy T, Landau S, Gavan K, Todd G, Whitaker W, et al. A practical comparison of two types of family intervention: an exploratory RCT of family day workshops and individual family work as a supplement to inpatient care for adults with anorexia nervosa. Eur Eat Disord Rev. 2012;20(2):142–50. https://doi.org/10.1002/erv.1076. | Same intervention delivered individually vs group based. |
|  | Salerno L, Rhind C, Hibbs R, Micali N, Schmidt U, Gowers S, et al. A longitudinal examination of dyadic distress patterns following a skills intervention for carers of adolescents with anorexia nervosa. Eur Child Adolesc Psychiatry. 2016;25(12):1337–47. https://doi.org/10.1007/s00787-016-0859-9. | Longitudinal sub-study of a multisite RCT. |
| Outcome of interest not assessed  (4) | Aarnio-Peterson CM, Le Grange D, Mara CA, Modi AC, Offenbacker North E, Zegarac M, Stevens K, Matthews A, Mitan L, Shaffer A. Emotion coaching skills as an augmentation to family-based therapy for adolescents with anorexia nervosa: A pilot effectiveness study with families with high expressed emotion. Int J Eat Disord. 2024;57(3):682–94. https://doi.org/10.1002/eat.24149. | No validated anxiety/depression scale in carers. |
|  | Breiner CE, Miller ML, Hormes JM. ARFID Parent Training Protocol: A randomized pilot trial evaluating a brief, parent-training program for avoidant/restrictive food intake disorder. Int J Eat Disord. 2021;54(12):2229–35. https://doi.org/10.1002/eat.23643. | Carer anxiety/depression outcomes not reported; ARFID-specific population. |
|  | Dimitropoulos G, Landers A, Freeman V, Novick J, Schmidt U, Olmsted M. A feasibility study comparing a web-based intervention to a workshop intervention for caregivers of adults with eating disorders. Eur Eat Disord Rev. 2019;27(6):641–54. https://doi.org/10.1002/erv.2678. | No validated anxiety/depression scale in carers. |
|  | Spettigue W, Maras D, Obeid N, Henderson KA, Buchholz A, Gomez R, Norris ML. A psycho-education intervention for parents of adolescents with eating disorders: a randomized controlled trial. Eat Disord. 2015;23(1):60–75. https://doi.org/10.1080/10640266.2014.940790. | No validated anxiety/depression scale in carers. |
| Overlapping populations  (2) | Magill N, Rhind C, Hibbs R, Goddard E, Macdonald P, Arcelus J, Morgan J, Beecham J, Schmidt U, Landau S, Treasure J. Two-year Follow-up of a Pragmatic Randomised Controlled Trial Examining the Effect of Adding a Carer’s Skill Training Intervention in Inpatients with Anorexia Nervosa. Eur Eat Disord Rev. 2016;24(2):122–30. https://doi.org/10.1002/erv.2422 | Overlapping populations with Hibbs et al., 2015 |
|  | Ruiz Á, Quiles Y, León-Zarceño E, Quiles MJ, Roncero M, Sanmartin R. Assessing the effectiveness and feasibility of the Experienced Carers Helping Others program in relatives of adolescents with eating disorders using an online application format with individual sessions. Fam Process. 2024;63(4):2151–75. https://doi.org/10.1111/famp.13049. | Overlapping populations with Ruiz et al., 2026 |
| Unsuitable population  (1) | Jacobi C, Hütter K, Völker U, Möbius K, Richter R, Trockel M, Jones Bell M, Lock J, Taylor CB. Efficacy of a Parent-Based, Indicated Prevention for Anorexia Nervosa: Randomized Controlled Trial. J Med Internet Res. 2018;20(12):e296. https://doi.org/10.2196/jmir.9464. | Population not of interest: at-risk (screening) sample; no clinical AN diagnosis |

ARFID = avoidant/restrictive food intake disorder; AN = anorexia nervosa.
