# Supplementary material for: Mental health interventions for carers of individuals with eating disorders: a systematic review of randomized controlled trials assessing efficacy on anxiety and depressive symptoms
Source: J Eat Disord. 2026 Jul 14;14:235. doi: 10.1186/s40337-026-01705-8 (PMC13647608; doi:10.1186/s40337-026-01705-8)
Supplement: Supplementary file 2 [file 40337_2026_1705_MOESM2_ESM.docx]

**Table A.2.** Characteristics of interventions.

| **Intervention type** | **Study** | **Protocol/Duration** | **Experimental group intervention** | **Control group condition** |
| --- | --- | --- | --- | --- |
| **Structured CIMM-based interventions** | Cardi et al., 2024 | Guided self-help: Digital platform + 8 group sessions; FU 12 and 18 months.  Guide: Trained facilitators. | ECHOMANTRA (guided) + TAU: Digital program combining ECHO and MANTRA; included workbook, recovery-oriented videos, and facilitated groups (patients, carers, joint). | TAU: Evidence-based treatment (NICE-guided), structured activities, medical reviews, and carer involvement. |
|  | Goddard et al., 2011 | Guided self-help: Book + 5 DVDs + 3 phone coaching sessions; FU 3 months.  Guide: Carer + senior clinical nurse. | ECHO (guided): Book + 5 DVDs (3 theoretical, 2 practical) + 3 coaching calls; motivational interviewing style and behaviour-change strategies. | ECHO (self-help): Book + 5 DVDs without additional coaching. |
|  | Hibbs et al., 2015 | Guided self-help: Book + 5 DVDs + 5–10 coaching calls (40 min each); FU 6 and 12 months.  Guide: Coaches, 64% skilled in motivational interviewing. | ECHO (guided) + TAU: Skills-based program (book + DVDs, role-plays, motivational interviewing strategies) plus coaching calls. | TAU: In-/day-patient care per NICE guidelines (CBT, IPT, psychodynamic therapy). |
|  | Hodsoll et al., 2017 | Unguided/guided self-help: Book + DVDs + 10 phone sessions; FU 12 months.  Guide: Experienced carers or postgraduate psychologists. | ECHO (unguided)/ECHO (guided) + TAU: Materials plus phone coaching (motivational interviewing); support provided by experienced carers and supervised clinicians. | TAU: Family-based therapy for adolescents per NICE; inpatient care if indicated. |
|  | Keshen et al., 2020 | Unguided self-help: ECHO DVD (4-week program); FU 3 months.  Guide: None (self-administered). | ECHO (unguided) + TAU using only ECHO DVD: 17 acted vignettes on effective strategies and supportive caring styles. | TAU: Intensive outpatient program (therapy groups, individual therapy, supervised meals, optional family sessions). |
|  | Ruiz et al., 2026 | Guided self-help: Eight weekly 60-min online sessions; FU 3, 6 and 12 months.  Guide: Assistant psychologists trained in ECHO. | ECHOMANTRA (guided) + TAU: Online adaptation of ECHO with tailored guidance. | TAU: National clinical guidelines + psychoeducational groups twice monthly. |
|  | Quiles Marcos et al., 2018 | Workshop: Six 2-hours group sessions over 3 months + FU workshop.  Guide: Psychologists trained in CCSTW. | CCSTW: Collaborative Care Skills Training Workshops covering motivational interviewing, functional analysis, and coping skills. | Psychoeducation: Six structured workshops with informational content only. |
|  | Sepulveda et al., 2019 | Workshop: Six 2-hours group sessions over 3 months + FU workshops.  Guide: Workshop facilitators. | CCSTW: Skills-based training in CBT, motivational interviewing, and emotional regulation strategies. | Psychoeducation: Adapted from Fairburn’s curriculum; illness-focused education for carers. |
| **CBT-based interventions** | Grover et al., 2011 | Guided self-help: Eight online modules + clinician guidance (≤20 min/week); 4-months program; FU 6 months.  Guide: Clinician. | OAO-G: Guided Overcoming Anorexia Online (web-based CBT system with systemic elements, self-monitoring, and coping skills). | Beat Support: Helpline, email support, moderated message board, and peer groups. |
|  | Hoyle et al., 2013 | Guided self-help: Seven online modules + workbooks; 7-week program; FU 3 months.  Guide: Masters-level trainee psychologist | OAO-G: Guided OAO (weekly email/phone support by trainee psychologist). | OAO-NoG: Same modules without guidance. |
| **Other interventions** | Quadflieg et al., 2017 | Unguided self-help + psychotherapy: 5 DVDs; baseline and 3-months post-discharge assessment.  Guide: None (DVD self-administered); optional psychotherapy/clinical counselling. | EG: Video-based CIMM intervention: Skills training program for carers during patient inpatient stay  EG+ : plus psychotherapy or clinical  counselling. | CG: TAU: Standard inpatient care with optional professional support  CG+ : plus psychotherapy or clinical  counselling. |
|  | McEvoy et al., 2019 | Workshop: Two 2.5-h sessions, 1 week apart.  Guide: Clinical psychologist. | A brief, face-to-face version of The New Maudsley Method, a psychoeducation and communication skills intervention derived from CIMM. | Waitlist. |

CG = control group; EG = experimental group, FU = follow up.

CIMM = Cognitive Interpersonal Maintenance Model; CBT = Cognitive Behavioural Therapy; IPT = Interpersonal psychotherapy; ECHO = Experienced Carers Helping Others; ECHOMANTRA = Experienced Carers Helping Others + Maudsley Model of Anorexia Nervosa Treatment for Adults; OAO-G = Overcoming Anorexia Online; OAO-NoG = Overcoming Anorexia Online No guidance; CCSTW = Collaborative Care Skills Training Workshops; TAU = Treatment as usual.
